# Lipid Storage and Autophagy in Melanoma Cancer Cells

**DOI:** 10.3390/ijms18061271

**Published:** 2017-06-15

**Authors:** Claudia Giampietri, Simonetta Petrungaro, Martina Cordella, Claudio Tabolacci, Luana Tomaipitinca, Antonio Facchiano, Adriana Eramo, Antonio Filippini, Francesco Facchiano, Elio Ziparo

**Affiliations:** 1 Department of Anatomy, Histology, Forensic Medicine and Orthopedics, Sapienza University of Rome, Rome 00161, Italy; simonetta.petrungaro@uniroma1.it (S.P.); luana.tomaipitinca@uniroma1.it (L.T.); antonio.filippini@uniroma1.it (A.F.); elio.ziparo@uniroma1.it (E.Z.); 2 Department of Oncology and Molecular Medicine Istituto Superiore di Sanità, ISS, Rome 00161, Italy; martina.cord87@gmail.com (M.C.); claudiotabolacci@tiscali.it (C.T.); d.eramo@libero.it (A.E.); francesco.facchiano@iss.it (F.F.); 3 Istituto Dermopatico dell’Immacolata IDI-IRCCS, Rome 00167, Italy; antoniofacchiano@gmail.com

**Keywords:** stem cells, autophagy, lipids, melanoma

## Abstract

Cancer stem cells (CSC) represent a key cellular subpopulation controlling biological features such as cancer progression in all cancer types. By using melanospheres established from human melanoma patients, we compared less differentiated melanosphere-derived CSC to differentiating melanosphere-derived cells. Increased lipid uptake was found in melanosphere-derived CSC vs. differentiating melanosphere-derived cells, paralleled by strong expression of lipogenic factors Sterol Regulatory Element-Binding Protein-1 (SREBP-1) and Peroxisome Proliferator-Activated Receptor-γ (PPAR-γ). An inverse relation between lipid-storing phenotype and autophagy was also found, since microtubule-associated protein 1A/1B-Light Chain 3 (LC3) lipidation is reduced in melanosphere-derived CSC. To investigate upstream autophagy regulators, Phospho-AMP activated Protein Kinase (P-AMPK) and Phospho-mammalian Target of Rapamycin (P-mTOR) were analyzed; lower P-AMPK and higher P-mTOR expression in melanosphere-derived CSC were found, thus explaining, at least in part, their lower autophagic activity. In addition, co-localization of LC3-stained autophagosome spots and perilipin-stained lipid droplets was demonstrated mainly in differentiating melanosphere-derived cells, further supporting the role of autophagy in lipid droplets clearance. The present manuscript demonstrates an inverse relationship between lipid-storing phenotype and melanoma stem cells differentiation, providing novel indications involving autophagy in melanoma stem cells biology.

## 1. Introduction

Melanoma is one of the most aggressive cancers and it has an increasing incidence worldwide [1]. Similar to other tumors, melanoma contains cancer stem cells (CSC). Such cells are able to grow as melanospheres making their isolation and indefinite growth from metastatic melanomas possible [2]. CSC represent the driving force within the tumor because they are able to produce all cell types found in the original tumor and are responsible for metastatic dissemination [3]. CSC represent a relevant therapeutic target since they are usually resistant to conventional chemotherapy and radiotherapy [4]; knowing in detail CSC biology is therefore crucial for planning effective therapeutic approaches. For such reasons, signaling pathways specifically activated in CSC but not in the bulk tumor are particularly relevant to identify novel therapeutically important targets.

Lipid levels within the cells appear to have a role in oncogenesis since inhibition of lipogenic enzymes (such as fatty acid synthase and acetyl-CoA carboxylase) is able to counteract growth of different tumors [5,6]. The link between altered lipid metabolism and oncogenesis is well accepted; nevertheless, lipid-related differences between CSC and bulk cells in the tumor are still a poorly investigated field. In melanoma, the metabolomic lipid profile of CSC may be different from the corresponding profile of bulk tumor cells [7]. Furthermore, in colon cancer, prostate cancer and melanoma, a correlation between high lipids content and metastatic potential was reported [8,9]. Few data are available regarding key molecular mechanisms underlying lipid accumulation. It is well recognized that cancer cells display increased biosynthesis of lipids through de novo fatty-acid synthesis, but little is known about additional mechanisms leading to the acquisition of a lipid-storing phenotype. Such phenotype has been demonstrated in colorectal cancer where high levels of lipid droplets (LD) correlate with CD133 antigen (also known as prominin-1) expression level and Wingless-type MMTV integration site family (Wnt)/β-catenin pathway activation [8], making LD abundance a marker of colorectal CSC. A LD-loaded phenotype has also been observed in glioblastoma and in lung cancer where LD accumulation is associated to increased spheroid-forming capacity [10]. In addition, metastatic potential of melanoma FEMX-1 cell line has been reported to correlate with both CD133 and LD levels [11]. LD are organelles containing neutral lipids (triglycerides and cholesteryl esters) surrounded by a phospholipid monolayer and coat proteins named perilipins [12]. Lipolysis of LD contributes to cellular metabolism, however, LD may also counteract lipotoxicity induced by potentially toxic intracellular fatty acid accumulation [13] or limit potentially toxic reactive oxygen species levels [14], avoiding peroxidation of polyunsaturated fatty acids[15]. Transcriptional activation of lipogenic genes such as Sterol Regulatory Element-Binding Protein-1 (SREBP-1) and Peroxisome Proliferator-Activated Receptor-γ (PPAR-γ) may be involved in LD accumulation. Their expression induces transdifferentiation of different cell types into adipocyte-like cells, thus accumulating triglycerides within LD [16]. Triglycerides stored inside LD may be hydrolyzed by cytosolic lipases or by lysosomal acidic lipases through the autophagic pathway. Autophagy, a highly conserved mechanism originally described in yeast, allows the turnover of cellular components and damaged organelles through double-membrane vesicles called autophagosomes, which fuse with lysosomes enabling autophagic substrates degradation by lysosomal enzymes and monomeric units recycle [16]. Mammalian Target Of Rapamycin (mTOR) is one of the main negative autophagy regulators since it can phosphorylate factors essential for the process initiation, such as UNC-51 Like Kinase (ULK1), leading to autophagy inhibition [17]. Conversely, AMP activated protein kinase (AMPK) is a positive autophagy regulator; in fact, AMPK phosphorylation has been shown to drive autophagy induction [18]. Under metabolic stress conditions, as a result of its activation, AMPK induces autophagy for energy homeostasis through degradation of macromolecular nutrients, such as glycogen and LD. During autophagy a cytosolic protein named microtubule-associated protein 1A/1B-Light Chain 3 (LC3-I) is processed to its phosphatidylethanolamine conjugate named LC3-II and recruited to the autophagosomal membranes. LC3-II quantification represents a suitable way to quantify autophagy since it increases under autophagy inducing conditions. When LD are sequestered into autophagosomes for their lysosomal breakdown, lipids are mobilized through a specific form of autophagy (also called lipophagy). Such process was originally described in hepatocytes, where lipophagy has been implicated in the regulation of cellular lipid content [19], and has also been shown in other cells types [20,21]. Remarkably, in clear cell renal cell carcinoma, lipophagy impairment leads to LD accumulation in the cytoplasm and correlates with carcinoma development, associated to bad prognosis [22].

Altogether, these data suggest that LD are cytosolic structures which deserve to be better explored in cancer models, as well as the cellular mechanisms associated to their accumulation. In the present study, we addressed such issue focusing on an in vitro model of melanoma CSC. Many reports demonstrate the CSC involvement in cancer initiation, progression and chemoresistance. Malignant melanoma CSC, similar to other CSC, generate continuously growing tumor [23]. As a CSC model, we used two cell lines established from human melanoma patients as previously described [2,24], namely Mel1 and Mel3. These cells were cultured as less differentiated melanosphere-derived CSC (MSC) and differentiating melanosphere-derived melanoma cells (PC).

We investigated the relationship between LD enrichment and autophagy. Interestingly, we found higher LD accumulation in melanosphere-derived cells as compared to differentiating melanoma cells. We also found differences between these cell types in the autophagic flux and in the activation of molecular regulators upstream this process. We concluded that LD enrichment characterizes melanoma CSC and that such LD-loaded phenotype correlates to autophagy down-regulation.

## 2. Results

### 2.1. Cells of Melanospheres Isolated from Metastatic Melanoma Display Abundant LD and Lipid Uptake as well as Strong Peroxisome Proliferator-Activated Receptor-γ (PPAR-γ) and Sterol Regulatory Element-Binding Protein-1 (SREBP-1) Expression

**Figure 1:**
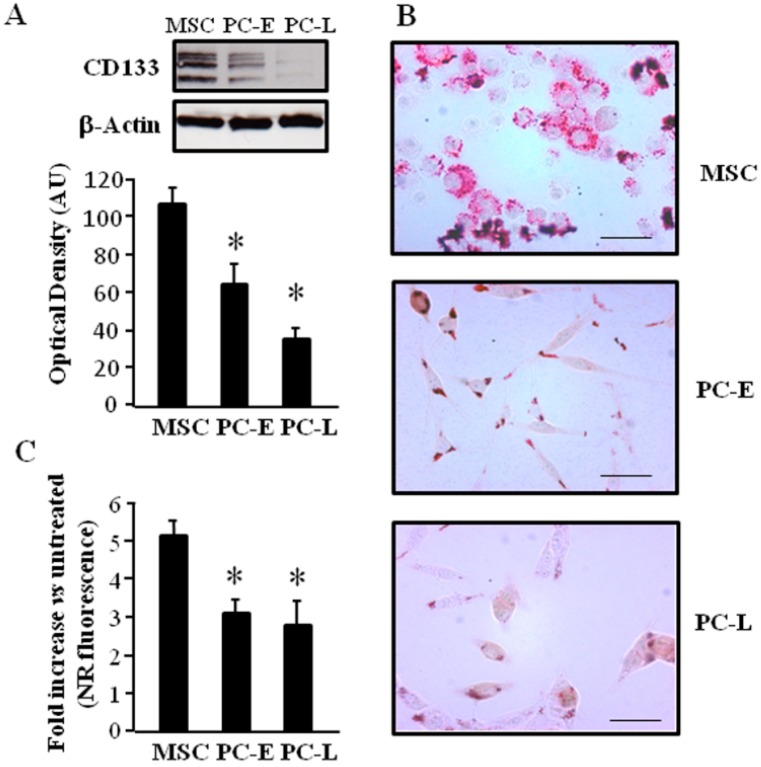
CD133 antigen (also known as prominin-1) expression and lipid-storing phenotype in melanosphere-derived cells. (**A**) CD133 marker is up-regulated in melanosphere-derived cancer stem cells (MSC). MSC and differentiating melanosphere-derived melanoma cells Early and Late (PC-E and PC-L) were lysed; protein samples were subjected to Western blot analyses and CD133 was detected. Western blot panel is representative of three independent experiments and reveal CD133 progressive down-regulation in melanoma cells undergoing progressive differentiation. β-Actin was used as a loading control. Densitometric analyses of CD133 relative to β-Actin in three independent experiments are presented in the graph as the mean ± s.d. expressed as Arbitrary Units (AU). * indicates *p* ≤ 0.05 vs. MSC; (**B**) Lipid Droplets (LD) staining is higher in MSC. MSC, PC-E and PC-L were stained with Oil Red O and then observed by bright-field microscopy. The images shown are representative of three independent experiments and reveal reduction in LD color intensity with melanoma cell differentiation. Bar corresponds to 35 μm; (**C**) MSC display increased lipid up-take. Increase of Nile Red (NR)-stained neutral lipids was evaluated by flow-cytometry after oleic acid treatment in MSC, PC-E and PC-L and reported in the graph as FL2 fluorescence fold increase vs. untreated cells. The data shown are the mean ± s.d. of three independent experiments. * indicates *p* ≤ 0.05 vs. MSC.

**Figure 2:**
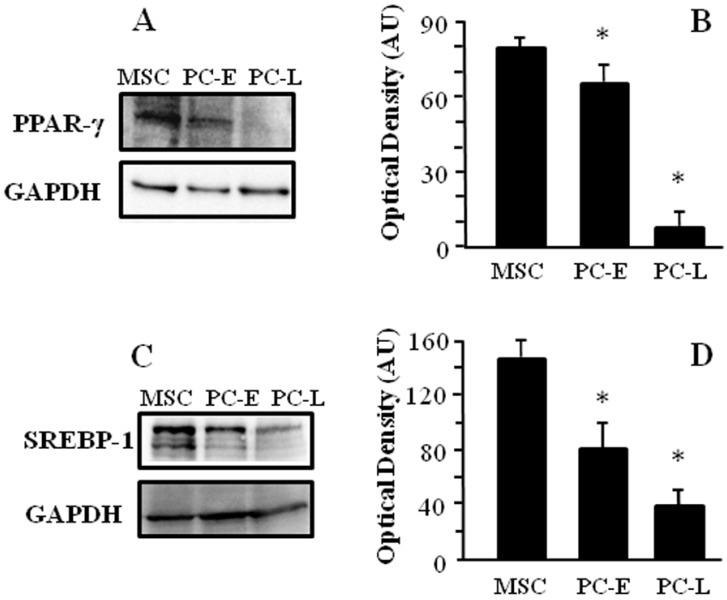
MSC express higher PPAR-γ and SREBP-1 levels as compared to PC-E and PC-L. MSC, PC-E and PC-L were lysed and protein samples were subjected to Western blot analyses using Glyceraldehyde 3-Phosphate Dehydrogenase (GAPDH) as a loading control. Representative panels of: PPAR-γ (**A**); and SREBP-1 (**C**) are shown. Densitometric analyses of: PPAR-γ (**B**); and SREBP-1 (**D**) relative to GAPDH in three independent experiments are presented in the graph as the mean ± s.d. expressed as Arbitrary Units (AU). PPAR-γ and SREBP-1 show down-regulation in melanoma cells undergoing progressive differentiation. * indicates *p* ≤ 0.05 in PC vs. MSC.

Lipogenic pathways are upregulated in different experimental cancer models, thus we investigated the attitude to accumulate lipids within LD both in melanosphere-derived CSC (MSC) and in melanosphere-derived early (PC-E) and late (PC-L) differentiating cells. We first analyzed the stemness marker CD133 [25] protein expression by western blot in total cell lysates and found higher level in MSC as compared to PC-E and PC-L as shown in Figure 1A. We then investigated if, similar to colorectal cancer, CD133 expression level correlates with LD abundance [8]. To this aim, we stained cells with Oil Red O, which allows selective detection of neutral lipids by light microscopy. MSC were found to be mostly characterized by multiple LD occupying the entire cytoplasm, while differentiating cells at either culture time, i.e., PC-E and PC-L, display moderate number or few LD mainly located at the cell periphery (Figure 1B). Addition of exogenous fatty acids is known to further stimulate LD formation [26]; we then investigated if LD staining in our experimental model increases upon fatty acid addition. MSC, PC-E and PC-L were treated with oleic acid and Nile Red-stained neutral lipids contained inside LD were quantified by flow cytometry. Higher fold elevation of neutral lipids staining after oleic acid treatment was found in MSC as compared to PC-E and PC-L, as shown in Figure 1C. Expression of transcription factors involved in lipid storage was then investigated. PPAR-γ and SREBP-1 proteins have been associated with fatty droplets accumulation [27,28,29]. We found higher level of both PPAR-γ and SREBP-1 in MSC as compared to PC-E and PC-L, as shown in Western blot experiments (Figure 2). We therefore concluded that melanosphere-derived melanoma cells display a lipid-storing phenotype.

### 2.2. MSC Display Lower Autophagic Flux as Compared to Differentiating Melanoma Cells

**Figure 3:**
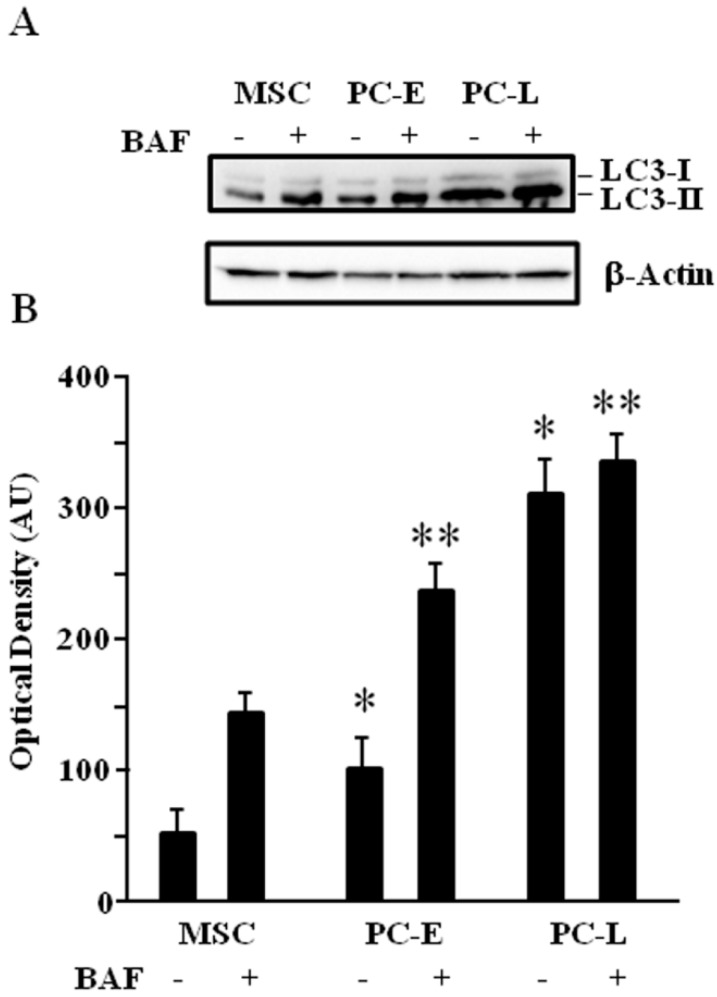
LC3-II increases in melanoma cells undergoing progressive differentiation revealing autophagic flux up-regulation. (**A**) MSC, PC-E and PC-L were cultured in the presence or absence of the lysosomal degradation inhibitor bafilomycin A1 (BAF). They were lysed and protein samples were subjected to Western blot analysis. LC3-II increase in differentiated melanoma cells, although evident in either case, is more evident after BAF treatment, thus demonstrating autophagic flux up-regulation as function of time of differentiation. Western blot panel is representative of three independent experiments. β-Actin was used as a loading control; (**B**) densitometric analysis of LC3-II relative to β-Actin in three independent experiments is presented in the graph as the mean ± s.d. expressed as AU. BAF-treated MSC vs. untreated MSC display significant increase (*p* ≤ 0.05). Similarly, ΒΑF treated PC-E vs. untreated PC-E display significant increase (*p* ≤ 0.05). ** indicates *p* ≤ 0.05 in BAF-treated PC vs. BAF-treated MSC. * indicates *p* ≤ 0.05 in untreated PC vs. untreated MSC.

LD have been shown to act as substrates of autophagy [19]. We then investigated if the different attitude to accumulate lipids in MSC as compared to PC-E and PC-L may correlate with a different autophagic flux, by measuring the membrane-bound LC3 (LC3-II) as previously reported [30]. LC3-II turnover may be too fast to be correctly assessed as expression change. Bafilomycin A1 has been reported to circumvent such problem [19]; it is an inhibitor of autophagosome and lysosome fusion, a late step of the process, and blocks LC3-II degradation and loss. Figure 3 shows that, upon bafilomycin A1 treatment, LC3-II level is lower in MSC as compared to PC-E and PC-L. We therefore concluded that differentiation of melanosphere-derived melanoma cells is associated to autophagic flux increase.

### 2.3. MSC Display Different AMPK and mTOR Phosphorylation as Compared to PC-E and PC-L

**Figure 4:**
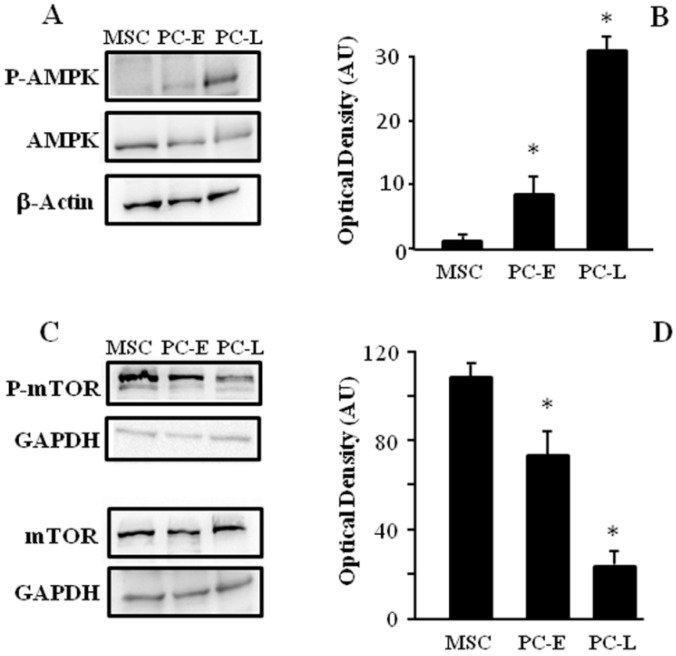
AMPK and mTOR phosphorylation levels change with melanoma cell differentiation. MSC, PC-E and PC-L were lysed and protein samples were subjected to Western blot using β-Actin or GAPDH as a loading control. Representative panels of P-AMPK and AMPK (**A**); and P-mTOR and m-TOR (**C**) are shown; (**B**,**D**) densitometric analyses of P-AMPK/AMPK and P-mTOR relative to the corresponding loading control, respectively, reveal progressive P-AMPK/AMPK increase and progressive P-mTOR decrease as function of time of differentiation. Data are presented as the mean ± s.d. expressed as AU of three independent experiments. * indicates *p* ≤ 0.05 in PC vs. MSC.

Key regulators of the autophagy process were then analyzed to elucidate molecular players underlying the observed differences in autophagic flux. Autophagy is often induced by AMPK, thus we investigated AMPK activation in MSC and PC. The phosphorylation of AMPK isoform α in Thr172, which is involved in AMPK activation, was found to be higher in PC-E and PC-L as compared to MSC as shown in Figure 4A,B.

We then investigated phosphorylation of mTOR in Ser2448 as a biomarker of mTORC complex-1 activation, since autophagy is related to inactivation of mTOR complex-1. mTOR phosphorylation in Ser2448 was found to be reduced in PC-E and PC-L cells as compared to MSC (Figure 4C,D), further confirming increased autophagic flux in differentiating melanoma cells as compared to melanosphere cells.

### 2.4. LD Co-Localization with Autophagosomes in Melanoma Cells

**Figure 5:**
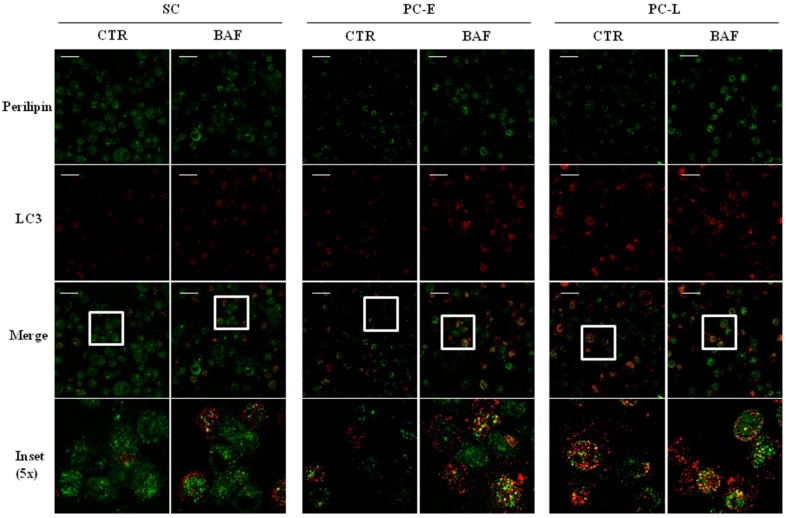
LC3-stained autophagosome spots co-localize with perilipin-stained LD suggesting LD clearance through autophagy. Immunofluorescence for perilipin (**green**) or LC3 (**red**) was performed on MSC, PC-E and PC-L, cultured in the absence (CTR) or presence of the lysosomal degradation inhibitor bafilomycin A1 (BAF). Yellow spots, indicating LC3-perilipin co-localization, increase as function of differentiation and are more evident in BAF-treated cells. Representative images of three experiments. All images were taken at the same magnification Bar corresponds to 60 μm. Insets correspond to further 5× enlargement.

In order to further investigate autophagy involvement in LD clearance, all three cell types were treated with bafilomycin A1 and then analyzed by immunofluorescence of the autophagosome marker LC3 and perilipin, a protein involved in LD coating. Representative immunofluorescence experiments are shown in Figure 5. As reported, bafilomycin A1 inhibits autophagosome/lysosome fusion and is used to better appreciate autophagosome staining [30]. Upon bafilomycin A1 treatment, we observed, as expected, increased punctuated LC3 signal as compared to the controls in all three cell types, confirming western blot results shown in Figure 3A. Furthermore, we found a more frequent co-localization of perilipin and LC3 in PC-E and PC-L, as compared to MSC, after bafilomycin A1 treatment. These data indicate that LD clearance may be blocked by bafilomycin A1, supporting that it may occur at least in part via autophagic flux. This finding strongly suggests that LD loss may be the result of enhanced autophagy.

Altogether, such data led us to hypothesize that higher LD accumulation observed in MSC (Figure 1B,C) may be the result of reduced autophagy found in MSC as compared to differentiating melanoma cells (Figure 3 and Figure 4).

## 3. Discussion

In the present study, we demonstrate a lipid storing-phenotype with lipid droplets (LD) accumulation in a stem cells melanoma model (MSC). LD are organelles involved in excess lipids storage and are surrounded by amphipathic lipids and coating proteins. We also show that such lipid storage is reduced in PC-E and PC-L, i.e., differentiated melanoma cells, and we relate such reduction to autophagy increase.

LD enrichment has been previously described in colorectal cancer stem cells. These cells express CD133 and activate Wnt pathway; they show high LD levels [8] and this represents their specific morphological signature correlating with high tumorigenic potential. Interestingly, in MSC, we found, similar to colorectal cancer stem cells, a positive relation between CD133 expression and LD accumulation In addition to their function in cellular lipid homeostasis, LD seem to protect cancer cells from reactive oxygen species toxicity [31] as well as to be relevant in the temporary sequestration of proteins such as histones, enzymes involved in purine synthesis and viral capsids [32]. In different cancer models lipid storage has been reported as a possible consequence of hypoxia and related to fatty acid uptake [31]. Some transcription factors have been involved in LD biogenesis such as SREBP and PPAR, which regulate the expression of genes participating in fatty acid uptake and consequent lipid accumulation [33]. These findings have been confirmed by our study regarding higher expression of SREBP-1 and PPAR-γ in MSC and lower levels in the differentiating PC-E and PC-L. Since various saturated or unsaturated long-chain fatty acids such as linoleic and oleic acid are important sources for LD formation, the effect of oleic acid treatment was evaluated in our experimental model. We found that MSC display higher oleic acid uptake than PC-E and PC-L and this phenotype parallels with higher LD accumulation observed in MSC.

On the other hand, LD degradation has been shown to be autophagy-related [20]. Autophagy may therefore clear LD and mobilize their accumulated lipids but the relative contribution of this pathway to lipolysis in cancer stem cell models has never been investigated. In the present study we addressed this issue and found that an increase of autophagic flux, evaluated through LC3-II level analysis in the presence or absence of bafilomycin A1, parallels the reduction of LD storage during melanoma stem cells differentiation. Such autophagy increase in PC as compared to MSC has also been confirmed by evaluating the phosphorylation level of proteins involved in autophagic activity (i.e., AMPK and mTOR) whose modulation depending on the differentiation level was found to correlate with autophagy stimulation.

According to our hypothesis, autophagy might be involved in LD degradation in PC, thus explaining reduced LD staining. Consistently with this, we found co-localization between LC3 stained autophagosomes spots and perilipin dots, particularly when autophagic flux is blocked by bafilomycin A1 treatment and in differentiating PC, indicating LD uptake into autophagosomes obtained in different experimental models, for instance in hepatocytes, where autophagy has been demonstrated to be responsible for LD degradation [20]. Remarkably, in hepatocytes, a bidirectional relation has been shown between autophagy and lipid stores. In fact, hepatic lipid accumulation has been demonstrated to decrease autophagic activity in high fat diet-fed mouse liver. In this model, a defect in autophagosome/lysosome fusion, possibly due to lipid alterations in autophagosomes or lysosomes, leads to increased lipid retention [19,20]. We are currently further investigating this issue in MSC.

The role of autophagy in cancer is quite debated. Thanks to its ability to recycle cellular components it has been considered a mechanism favoring cancer cells, which generally have higher energy requirements than normal counterparts. On the other hand, defects in autophagic genes have been linked with increased tumorigenesis, thus supporting its tumor-suppressing function [34]. The present manuscript describes a relation between lipid-storing phenotype, autophagic flux level and melanoma stem cells differentiation, and provides indications suggesting that autophagy may be involved in melanoma stem cells biology. The role of autophagy in regulating pluripotency and differentiation of CSC is largely unknown. A recent study by Sharif and collaborators [35] demonstrates that a basal level of autophagy is crucial for the pluripotency of CSC (embryonal carcinoma cells (ECC)) and that increase or decrease in this autophagy basal level forces CSC toward differentiation and senescence. Moreover, the interplay between autophagy and differentiation has been also underlined in MCF-7 breast cancer cells [36] where autophagy activation is strictly linked to loss of mitotic and metastatic potential. Further studies are ongoing in order to investigate if, similar to ECC cells, an accurate modulation of autophagy basal level may maintain MSC in an undifferentiated state and interfere with their differentiation.

Our results support a novel role for fatty acid and lipid storage/recycling mechanisms in cancer cell stemness and cell destiny as suggested in recent studies involving other models. As recently pointed out, molecular mechanisms promoting LD accumulation have not been described yet in cancer cells, while LD biogenesis appears to have a key role in tumorigenesis [37]. The role of lipids in autophagy control is currently under investigation; remarkably LC3 lipidation has been identified as one of the key steps of autophagosome formation to control autophagosome membranes kinetics [38]. We believe the present study reveals novel mechanisms by which lipids and lipid intake relate with stem cell fate.

## 4. Materials and Methods

### 4.1. Cell Cultures

Data of the present study were collected from melanosphere-derived human melanoma cells and from two differentiating stages of melanosphere-derived human melanoma cells.

More in detail, melanoma CSC, namely Mel1 CSC, were obtained by surgical specimens as previously described and cultured as less differentiated melanospheres (MSC) in stem cell medium [2]. Briefly, after washing surgical specimens several times, their tissue dissociation was carried out. Recovered cells were cultured on flasks non-treated for tissue culture in order to reduce cell adherence and support their growth within undifferentiated tumor-spheres. Serum-free medium containing 50 μg/mL insulin, 100 μg/mL apo-transferrin, 10 μg/mL putrescine, 0.03 μM sodium selenite, 2 μM progesterone, 0.6% glucose, 5 mM HEPES, 0.1% sodium bicarbonate, 0.4% BSA, glutamine and antibiotics, dissolved in DMEM-F12 medium (Gibco-Invitrogen, Carlsbad, CA, USA) and supplemented with 20 ng/mL EGF and 10 ng/mL bFGF was used. It was replaced twice a week until cells started to grow forming floating aggregates. More-differentiated cells (PC) were obtained by growing melanospheres in Melanocyte Growth Medium (MGM4, Lonza, East Rutherford, NJ, USA) for 4 days (PC early, PC-E) or 8 days (PC late, PC-L). In PC-L cultures, growth medium was changed after four days.

Additional experiments were carried out on another melanoma-propagating cell line, namely Mel3.

Oleic acid (Sigma-Aldrich, Milano, Italy) 400µM was incubated for 24 h. Bafilomycin A1 (Sigma-Aldrich) 100 nM was added 4 h before cell harvesting.

### 4.2. Oil Red O Staining

Briefly, a stock oil red solution was prepared diluting 0.7 g Oil Red O with 200 mL isopropanol. A working dilution was then obtained by mixing 6 parts Oil Red O stock with 4 parts dH_2_O. MSC were harvested, smeared on slides by cytospin centrifugation. Cells were fixed with 10% formalin 5 min at room temperature. Then fresh formalin was added and incubated 1 h. After formalin removal, cells were washed with 60% isopropanol 5 min at room temperature. After isopropanol removal, oil red working solution was added for 10 min. Cells were then washed with H_2_O and analyzed immediately by light microscopy. The Axioskop 2 plus microscope (Carl Zeiss Microimaging, Inc., Milan, Italy) was used. Images were obtained at room temperature using AxioCamHRC camera (Carl Zeiss Microimaging, Inc.) by Axiovision software (version 3.1, Carl Zeiss Microimaging, Inc.).

### 4.3. Lipid Determination by Flow Cytometry

Lipid content was assessed by multi-parameter flow cytometry, in association with fluorescent dye Nile Red (NR) using Lipid Droplets Fluorescence Assay Kit (Cayman, Ann Arbor, MI, USA). Cells were stained with NR according to producer’s instructions. The NR fluorescence was determined using CyAn ADP flow cytometer equipped with a 488 nm, 20mW semiconductor laser. Upon excitation by the 488 nm laser, NR emits at 580 nm when dissolved in neutral lipids [39] which are detected by the FL2 (575/25) emission. For each sample, 150,000 cells were analyzed. Flow cytometry data were analyzed using the DeNovo Software FCS Express5.0 (DeNovo, Glendale, CA, USA).

### 4.4. Immunoblotting

Immunoblot analysis was carried out as previously described [40]. Membranes were probed using the following antibodies: anti-CD133 (Biorbyt, Cambridge, UK), anti-SREBP-1 and anti-GAPDH (Santa Cruz, Heidelberg, Germany), anti-p62 (Abcam, Cambridge, UK), anti-β-Actin-HRP (Sigma-Aldrich, Milano, Italy), anti-mTOR, anti-P-mTOR (Ser2448), anti-AMPK, anti-P-AMPK (Thr172), anti-LC3, anti-PPAR-γ (Cell Signaling, Danvers, MA, USA). Secondary antibodies were horseradish peroxidase-conjugated goat anti-mouse or anti-rabbit (Bio-Rad, Hercules, CA, USA). PVDF membranes (Amersham Bioscience, Piscataway, NJ, USA) were used for anti-LC3 immunoblot. Nitrocellulose membranes (Amersham Bioscience) were probed with all other antibodies.

### 4.5. Immunofluorescence

For immunofluorescence experiments, slides were prepared as described [41,42,43]. Briefly, MSC, PC-E and PC-L, were gently resuspended and laid on poly-lysine coated slides for 3 min at 37 °C. Cells were fixed for 10 min at room temperature with 4% paraformaldehyde (Sigma), permeabilized for 10 min with HEPES-buffered PBS (Sigma) containing 0.1% saponin (Sigma) and 3% BSA (Sigma), and stained with anti-LC3 mAbs (Nanotools, Teningen, Germany) and anti-perilipin (Cell Signaling), followed by goat anti-mouse isotype-specific Ab or goat anti-rabbit Ab labeled with Alexa Fluor 555 and 488 (Invitrogen), respectively. Slides were analyzed using a Leica laser confocal microscope TCS SP2 [44]. Images were processed with ImageJ software (open platform developed at NIH, Bethesda, MD, USA for scientific image analysis, https://imagej.nih.gov/ij/) and Leica LAS AF Lite software (version 4.0, Leica Microsystems, Milan, Italy, http://downloads.informer.com/leica-las-af-lite/download/).

### 4.6. Data Handling and Statistical Analysis

All experiments were performed at least three times. Data collected were averaged and expressed as mean ± standard deviation (s.d.). Unpaired two-tailed student’s *t*-test was carried out and significance was set at *p* ≤ 0.05.

## 5. Conclusions 

The present manuscript demonstrates an inverse relationship between lipid-storing phenotype and melanoma stem cells differentiation, providing novel indications involving autophagy in melanoma stem cells biology.

## References

[1] Singh S., Zafar A., Khan S., Naseem I. (2017). Towards therapeutic advances in melanoma management: An overview. Life Sci..

[2] Sette G., Fecchi K., Salvati V., Lotti F., Pilozzi E., Duranti E., Biffoni M., Pagliuca A., Martinetti D., Memeo L. (2013). Mek inhibition results in marked antitumor activity against metastatic melanoma patient-derived melanospheres and in melanosphere-generated xenografts. J. Exp. Clin. Cancer Res..

[3] Ailles L.E., Weissman I.L. (2007). Cancer stem cells in solid tumors. Curr. Opin. Biotechnol..

[4] Cojoc M., Mabert K., Muders M.H., Dubrovska A. (2015). A role for cancer stem cells in therapy resistance: Cellular and molecular mechanisms. Semin. Cancer Biol..

[5] Johnson D.L., Stiles B.L. (2016). Maf1, a new PTEN target linking RNA and lipid metabolism. Trends Endocrinol. Metab..

[6] Jones S.F., Infante J.R. (2015). Molecular pathways: Fatty acid synthase. Clin Cancer Res..

[7] Song M., Lee H., Nam M.H., Jeong E., Kim S., Hong Y., Kim N., Yim H.Y., Yoo Y.J., Kim J.S. (2016). Loss-of-function screens of druggable targetome against cancer stem-like cells. FASEB J..

[8] Tirinato L., Liberale C., Di Franco S., Candeloro P., Benfante A., La Rocca R., Potze L., Marotta R., Ruffilli R., Rajamanickam V.P. (2015). Lipid droplets: A new player in colorectal cancer stem cells unveiled by spectroscopic imaging. Stem Cells.

[9] Rappa G., Fargeas C.A., Le T.T., Corbeil D., Lorico A. (2015). Letter to the editor: An intriguing relationship between lipid droplets, cholesterol-binding protein CD133 and Wnt/β-catenin signaling pathway in carcinogenesis. Stem Cells.

[10] Menard J.A., Christianson H.C., Kucharzewska P., Bourseau-Guilmain E., Svensson K.J., Lindqvist E., Indira Chandran V., Kjellen L., Welinder C., Bengzon J. (2016). Metastasis stimulation by hypoxia and acidosis-induced extracellular lipid uptake is mediated by proteoglycan-dependent endocytosis. Cancer Res..

[11] Rappa G., Mercapide J., Anzanello F., Le T.T., Johlfs M.G., Fiscus R.R., Wilsch-Brauninger M., Corbeil D., Lorico A. (2013). Wnt interaction and extracellular release of prominin-1/CD133 in human malignant melanoma cells. Exp. Cell Res..

[12] Ward C., Martinez-Lopez N., Otten E.G., Carroll B., Maetzel D., Singh R., Sarkar S., Korolchuk V.I. (2016). Autophagy, lipophagy and lysosomal lipid storage disorders. Biochim. Biophys. Acta.

[13] Shatz O., Holland P., Elazar Z., Simonsen A. (2016). Complex relations between phospholipids, autophagy, and neutral lipids. Trends Biochem. Sci..

[14] Giampietri C., Starace D., Petrungaro S., Filippini A., Ziparo E. (2014). Necroptosis: Molecular signalling and translational implications. Int. J. Cell. Biol..

[15] Bailey A.P., Koster G., Guillermier C., Hirst E.M., MacRae J.I., Lechene C.P., Postle A.D., Gould A.P. (2015). Antioxidant role for lipid droplets in a stem cell niche of drosophila. Cell.

[16] Liu S., Wang Y., Wang L., Wang N., Li Y., Li H. (2010). Transdifferentiation of fibroblasts into adipocyte-like cells by chicken adipogenic transcription factors. Comp. Biochem. Physiol. A.

[17] Alers S., Loffler A.S., Wesselborg S., Stork B. (2012). Role of AMPK-mTOR-Ulk1/2 in the regulation of autophagy: Cross talk, shortcuts, and feedbacks. Mol. Cell. Biol..

[18] Kim J., Kundu M., Viollet B., Guan K.L. (2011). Ampk and mTOR regulate autophagy through direct phosphorylation of Ulk1. Nat. Cell. Biol..

[19] Dong H., Czaja M.J. (2011). Regulation of lipid droplets by autophagy. Trends Endocrinol. Metab..

[20] Singh R., Kaushik S., Wang Y., Xiang Y., Novak I., Komatsu M., Tanaka K., Cuervo A.M., Czaja M.J. (2009). Autophagy regulates lipid metabolism. Nature.

[21] Martinez-Vicente M., Talloczy Z., Wong E., Tang G., Koga H., Kaushik S., de Vries R., Arias E., Harris S., Sulzer D. (2010). Cargo recognition failure is responsible for inefficient autophagy in huntington’s disease. Nat. Neurosci..

[22] Xu G., Jiang Y., Xiao Y., Liu X.D., Yue F., Li W., Li X., He Y., Jiang X., Huang H. (2016). Fast clearance of lipid droplets through MAP1S-activated autophagy suppresses clear cell renal cell carcinomas and promotes patient survival. Oncotarget.

[23] Regad T. (2013). Molecular and cellular pathogenesis of melanoma initiation and progression. Cell. Mol. Life Sci..

[24] Tabolacci C., Cordella M., Turcano L., Rossi S., Lentini A., Mariotti S., Nisini R., Sette G., Eramo A., Piredda L. (2015). Aloe-emodin exerts a potent anticancer and immunomodulatory activity on BRAF-mutated human melanoma cells. Eur. J. Pharmacol..

[25] Madjd Z., Erfani E., Gheytanchi E., Moradi-Lakeh M., Shariftabrizi A., Asadi-Lari M. (2016). Expression of CD133 cancer stem cell marker in melanoma: A systematic review and meta-analysis. Int. J. Biol. Markers.

[26] Rohwedder A., Zhang Q., Rudge S.A., Wakelam M.J. (2014). Lipid droplet formation in response to oleic acid in Huh-7 cells is mediated by the fatty acid receptor FFAR4. J. Cell. Sci..

[27] Shieh Y.S., Chang Y.S., Hong J.R., Chen L.J., Jou L.K., Hsu C.C., Her G.M. (2010). Increase of hepatic fat accumulation by liver specific expression of hepatitis B virus X protein in zebrafish. Biochim. Biophys. Acta.

[28] Yokoyama C., Wang X., Briggs M.R., Admon A., Wu J., Hua X., Goldstein J.L., Brown M.S. (1993). SREBP-1, a basic-helix-loop-helix-leucine zipper protein that controls transcription of the low density lipoprotein receptor gene. Cell.

[29] Lee A.H., Scapa E.F., Cohen D.E., Glimcher L.H. (2008). Regulation of hepatic lipogenesis by the transcription factor XBP1. Science.

[30] Klionsky D.J., Abdelmohsen K., Abe A., Abedin M.J., Abeliovich H., Acevedo Arozena A., Adachi H., Adams C.M., Adams P.D., Adeli K. (2016). Guidelines for the use and interpretation of assays for monitoring autophagy (3rd edition). Autophagy.

[31] Bensaad K., Favaro E., Lewis C.A., Peck B., Lord S., Collins J.M., Pinnick K.E., Wigfield S., Buffa F.M., Li J.L. (2014). Fatty acid uptake and lipid storage induced by HIF-1α contribute to cell growth and survival after hypoxia-reoxygenation. Cell Rep..

[32] Welte M.A. (2007). Proteins under new management: Lipid droplets deliver. Trends Cell. Biol..

[33] Bozza P.T., Viola J.P. (2010). Lipid droplets in inflammation and cancer. Prostaglandins Leukot. Essent. Fatty Acids.

[34] Towers C.G., Thorburn A. (2016). Therapeutic targeting of autophagy. EBioMedicine.

[35] Sharif T., Martell E., Dai C., Kennedy B.E., Murphy P., Clements D.R., Kim Y., Lee P.W., Gujar S.A. (2017). Autophagic homeostasis is required for the pluripotency of cancer stem cells. Autophagy.

[36] Chakraborty A., Bodipati N., Demonacos M.K., Peddinti R., Ghosh K., Roy P. (2012). Long term induction by pterostilbene results in autophagy and cellular differentiation in MCF-7 cells via ROS dependent pathway. Mol. Cell. Endocrinol..

[37] Roy D., Mondal S., Khurana A., Jung D.B., Hoffmann R., He X., Kalogera E., Dierks T., Hammond E., Dredge K. (2017). Loss of HSulf-1: The missing link between autophagy and lipid droplets in ovarian cancer. Sci. Rep..

[38] Pavel M., Rubinsztein D.C. (2017). Mammalian autophagy and the plasma membrane. FEBS J..

[39] Andrade R., Leal R., Roseiro J., Reis A., da Silva T.L. (2012). Monitoring rhodosporidium toruloides ncyc 921 batch fermentations growing under carbon and nitrogen limitation by flow cytometry. World J. Microbiol. Biotechnol..

[40] Giampietri C., Petrungaro S., Conti S., Facchiano A., Filippini A., Ziparo E. (2015). c-Flip KO fibroblasts display lipid accumulation associated with endoplasmic reticulum stress. Biochim. Biophys. Acta.

[41] Faroudi M., Utzny C., Salio M., Cerundolo V., Guiraud M., Muller S., Valitutti S. (2003). Lytic versus stimulatory synapse in cytotoxic T lymphocyte/target cell interaction: Manifestation of a dual activation threshold. Proc. Natl. Acad. Sci. USA.

[42] Mancinelli R., Franchitto A., Glaser S., Meng F., Onori P., Demorrow S., Francis H., Venter J., Carpino G., Baker K. (2013). GABA induces the differentiation of small into large cholangiocytes by activation of Ca(2+) /CaMK I-dependent adenylyl cyclase 8. Hepatology.

[43] Francis H., Onori P., Gaudio E., Franchitto A., DeMorrow S., Venter J., Kopriva S., Carpino G., Mancinelli R., White M. (2009). H3 histamine receptor-mediated activation of protein kinase cα inhibits the growth of cholangiocarcinoma in vitro and in vivo. Mol. Cancer Res..

[44] Antonangeli F., Giampietri C., Petrungaro S., Filippini A., Ziparo E. (2009). Expression profile of a 400-bp Stra8 promoter region during spermatogenesis. Microsc. Res. Tech..

